# Body-mass index and long-term risk of sepsis-related mortality: a population-based cohort study of 0.5 million Chinese adults

**DOI:** 10.1186/s13054-020-03229-2

**Published:** 2020-08-31

**Authors:** Li Weng, Junning Fan, Canqing Yu, Yu Guo, Zheng Bian, Yuxia Wei, Ling Yang, Yiping Chen, Huaidong Du, Liang Chang, Weiwei Gong, Junshi Chen, Zhengming Chen, Bin Du, Jun Lv, Liming Li

**Affiliations:** 1 Medical Intensive Care Unit, Peking Union Medical College Hospital, Peking Union Medical College, Chinese Academy of Medical Sciences, Beijing, 100730 China; 2 Department of Epidemiology and Biostatistics, School of Public Health, Peking University Health Science Center, 38 Xueyuan Road, Beijing, 100191 China; 3 Chinese Academy of Medical Sciences, Beijing, China; 4 Medical Research Council Population Health Research Unit, University of Oxford, Oxford, UK; 5 Nuffield Department of Population Health, Clinical Trial Service Unit & Epidemiological Studies Unit (CTSU), University of Oxford, Oxford, UK; 6 Henan Center for Disease Control and Prevention, Henan, China; 7 Zhejiang Center for Disease Control and Prevention, Zhejiang, China; 8 China National Center for Food Safety Risk Assessment, Beijing, China; 9 Key Laboratory of Molecular Cardiovascular Sciences (Peking University), Ministry of Education, Beijing, China; 10 Peking University Institute of Environmental Medicine, Beijing, China

**Keywords:** Sepsis, Body-mass index, Waist circumference, Mortality, Cohort study

## Abstract

**Background:**

Sepsis represents a major worldwide healthcare burden. However, how body-mass index (BMI) is related to the long-term risk of sepsis-related mortality in low- and middle-income countries remains uncertain.

**Methods:**

We examined the associations of sepsis-related mortality with both baseline BMI and waist circumference (WC) using data from China Kadoorie Biobank, a prospective cohort recruited during 2004–2008 and followed up to December 2016. After excluding participants with chronic obstructive pulmonary disease, tuberculosis, cancer, heart disease, and stroke, and omitting the first 3 years of follow-up, 440,763 participants remained for analysis.

**Results:**

During a median follow-up of 10.0 years, 1957 sepsis-related deaths (3,134,870 person-years) were included for analysis. Compared with reference BMI of 22.5 to < 25.0 kg/m^2^, the multivariable-adjusted hazard ratios (HRs) for sepsis-related mortality were 2.42 (95% CIs 2.07–2.84) for BMI of < 18.5, 1.59 (1.36–1.85) for 18.5 to < 20.0, 1.21 (1.06–1.38) for 20.0 to < 22.5, 0.97 (0.83–1.13) for 25.0 to < 27.5, 0.98 (0.80–1.21) for 27.5 to < 30.0, and 1.22 (0.93–1.60) for ≥ 30.0 kg/m^2^. Further adjustment for WC led to slightly augmentation of the effect size for the lower BMI groups and null association in the obese group. In the association analysis between WC and sepsis-related mortality, compared with the middle quintile group, only the highest quintile group showed an increased risk of sepsis-related mortality after adjusted for BMI (HR = 1.54; 95% CI 1.28–1.84).

**Conclusions:**

Underweight, lower normal weight, and abdominal obesity are associated with increased future risk of sepsis-related mortality over 10 years in the Chinese population. The double burden of underweight and obesity indicates a heavy sepsis burden faced by low- and middle-income countries.

## Introduction

Sepsis represents a major healthcare burden in both high-income countries (HICs) and low- and middle-income countries (LMICs) [1–4], with approximately 70% of sepsis occurring in the community [2]. Despite improved understanding of pathogenesis, treatment of sepsis is mainly supportive, and sepsis-related mortality remains up to 40% [5–7]. In a recent study of 300 sepsis-related deaths from 6 US hospitals, 88% were judged unlikely to be preventable even through better medical care [8]. As a result, investigation of potential risk factors for sepsis and sepsis-related mortality may facilitate sepsis prevention and screening of at-risk patients for early recognition and treatment, and further reduce the burden of sepsis.

Observational studies in septic patients examining the association between body-mass index (BMI) at hospital admission and sepsis-related death suggested protective effects of overweight on survival, while conflicting findings were reported for underweight and obesity [9–11]. However, these studies were subject to reverse causality [12, 13], inadequate control of confounding by smoking [13], and estimation errors of height and weight [14]. Also, such studies that enrolled hospitalized patients precluded the generalization of their findings to the impact of BMI on the long-term risk of sepsis-related death.

Previous population-based prospective cohort studies assessing the association between BMI and long-term risk of sepsis-related death were only available in HICs, with two studies focusing on specific infections (such as pneumonia [15] and bloodstream infection [16]) in general population, and another study reporting sepsis-related death in a cohort of walkers and runners who were exercising at substantially higher levels than general population [17]. Whether these findings can be generalized to the population in LMICs remains uncertain. Over the past several decades, the dramatic nutrition transition in LMICs has led to a double burden of malnutrition characterized by coexisting underweight and obesity [18], the impact of which on sepsis burden in LMICs merits further investigation.

This study aimed to evaluate the association between BMI and long-term risk of sepsis-related mortality over 10 years using a population-based prospective cohort of 0.5 million Chinese adults. Further analysis with waist circumference (WC) would help clarify the potential role of abdominal obesity in the risk of sepsis-related mortality.

## Materials and methods

### Study population

Detailed information about the China Kadoorie Biobank (CKB) study design and implementation has been reported previously [19, 20]. Briefly, 512,715 men and women aged 30–79 years were recruited from 10 geographically diverse (five urban and five rural) areas across China during 2004–2008. A standardized laptop-based questionnaire survey and physical measurements were undertaken for each participant, and a 10-ml random blood sample was collected. All participants were followed up for morbidity and mortality since they enrolled in the study.

The study was approved by the Ethical Review Committee of the Chinese Center for Disease Control and Prevention (Beijing, China) and the Oxford Tropical Research Ethics Committee, University of Oxford (UK). All participants provided written informed consent.

### Assessment of anthropometric measures

At baseline, trained staff took anthropometric measurements according to a standard protocol while participants were wearing light clothes and no shoes. Standing height was measured using a stadiometer. Weight was measured using a body composition analyzer (TANITA-TBF-300 GS; Tanita Corporation). WC at midway between the lowest rib and the iliac crest, and hip circumference (HC) at the maximum circumference around the buttocks were measured using a soft non-stretchable tape.

BMI was calculated as measured weight in kilograms divided by the standing height in meters squared. We divided BMI into seven groups in referring to previous study [12]: < 18.5 (underweight), 18.5 to < 20.0 (low-normal weight), 20.0 to < 22.5 (mid-normal weight), 22.5 to < 25.0 (high-normal weight, the reference group for analysis), 25.0 to < 27.5 (low overweight), 27.5 to < 30.0 (high overweight), and ≥ 30.0 (obesity). WC was categorized into five groups according to sex-specific quintiles, with the middle quintile (Q3) as the reference group.

### Assessment of covariates

At baseline, covariates collected by questionnaire included socio-demographic characteristics (age, sex, region, education, occupation, household income, and marital status), dietary and lifestyle factors (smoking status, alcohol consumption, physical activity, intakes of fresh fruits, vegetables, and red meat), personal medical history, and self-rated health status. Prevalent diabetes at baseline was defined as measured fasting blood glucose ≥ 7.0 mmol/l, measured random blood glucose ≥ 11.1 mmol/l, or self-reported diagnosis of diabetes.

### Ascertainment of outcomes

The mortality data of CKB during follow-up was based on information from local disease surveillance points (DSPs) system, a vital registry system established since 1978 [21]. Causes of death were derived mainly from the official death certificate, or supplemented, if necessary, by reviewing medical records or conducting standardized verbal autopsy procedures [22]. Multiple ways have been employed to maximize death ascertainment, including electronic linkage to the local health insurance records, annual checks against residential records, or directly contacting participants if necessary. More information on incident non-fatal diseases was collected by ongoing linkage to established disease registries and national health insurance claim databases.

All causes of mortality and morbidity were coded by trained staff who were blinded to the baseline information using International Statistical Classification of Diseases and Related Health Problems 10th Revision (ICD-10). For the present study, the primary outcome was sepsis-related mortality. Specifically, death attributed to any infections potentially related to sepsis was ascertained by the presence of any related infection code in part I or II of the certificate of death, i.e., immediate or underlying causes of death (detailed ICD-10 codes shown in Additional file 1: Table S1) [1]. Otherwise, death was classified as non-sepsis-related mortality.

The strategy of the ICD-10 coding algorithm for identifying sepsis-related mortality has been validated in our previous study of 3849 decedents [1]. In the CKB study, there is an ongoing process of retrieving medical records of major chronic disease for adjudication. Among the sepsis-related deaths and non-sepsis-related deaths by the ICD-10 criteria, we selected 119 and 103 patients (proportionally from 10 study areas) respectively according to the following criteria: those had retrieved medical records, and the death occurred within 1 week of this hospitalization or discharge. One senior intensivist validated these death cases by manual review of medical records. A modified sepsis definition according to a previously validated clinical surveillance definition [23] based on sepsis-3 criteria [24] was used as a reference. Sepsis was confirmed if a patient had documented infection concurrent with at least one acute organ dysfunction before death. Acute organ dysfunction was defined as follows: (1) hypotension or initiation of vasopressor, (2) hypoxemia requiring mechanical ventilation, (3) serum lactate > 2.0 mmol/l, (4) oliguria or significantly increased creatinine requiring renal replacement therapy, (5) platelet count significantly decreased from baseline and < 100 cells/μl, and (6) total bilirubin doubled from baseline. It turned out that 84.6% of selected sepsis-related mortality was confirmed with our modified sepsis definition and 86.4% of non-sepsis-related mortality did not meet the definition.

### Statistical analysis

In the current study, we excluded participants with missing or implausible/extreme values of BMI and weight (BMI < 15.0 or > 50.0 kg/m^2^, or weight < 30 kg; *n* = 454). To minimize reverse causation due to effects of pre-existing diseases on baseline BMI, the prespecified primary analysis excluded participants with self-reported doctor-diagnosed stroke (*n* = 8884), ischemic heart disease (*n* = 15,472), cancer (*n* = 2578), chronic obstructive pulmonary disease (COPD; *n* = 37,055), or tuberculosis (*n* = 7659) at baseline. We also excluded the first 3 years of follow-up (*n* = 5920), since known or unknown diseases at baseline that predispose participants to death over the next few years could lead to lower BMI at baseline, i.e., reverse causation. After these exclusions, 440,763 participants remained for analysis (Additional file 1: Figure S1). Due to a significantly higher prevalence of smoking in Chinese men than in women, an analysis restricted to never smokers, a better control for residual confounding by smoking, may lead to results that are more applicable to women. Therefore, the results of never smokers are presented but not as the primary analysis.

Participants contributed to the analysis from baseline (2004–2008) until the date of death, loss to follow-up, or December 31, 2016, whichever occurred first. By the end of 2016, less than 1% of all CKB participants were lost to follow-up, that is, participants have moved their permanent registered residence out of the jurisdiction of the Regional Coordinating Center. The Kaplan-Meier survival curve was plotted to compare survival probabilities by different BMI groups. The Cox proportional hazards model was used to estimate hazard ratios (HRs) and 95% confidence intervals (95% CIs) for the association of mortality with BMI or WC, with age as the time scale and stratified jointly by age at baseline in 5-year intervals, sex, and 10 study areas. The proportional hazards assumptions for the Cox model were assessed using Schoenfeld residuals and were satisfied. Multivariable models were adjusted for education; alcohol consumption; physical activity; intake frequency of fresh fruits, vegetables, and red meat; and smoking status. In the analysis of WC and sepsis-related mortality, we additionally adjusted for HC that allows a more precise estimation of the detrimental effects of visceral adipose tissue measured by WC [25]. Further analyses were conducted by mutually adjusting for BMI and WC.

To assess the robustness of the main results, several sensitivity analyses were performed. We additionally adjusted for baseline household income, occupation, and marital status; additionally adjusted for incident diseases during follow-up, including COPD, pneumonia, respiratory diseases other than COPD or pneumonia, tuberculosis, cancer, and diabetes; excluded participants with diabetes at baseline; and excluded those with self-rated poor health status at baseline. Subgroup analyses were performed by age (< 65 or ≥ 65 years old), sex (men or women), study area (rural or urban), and smoking status (current smokers or non-current smokers; former smokers who had stopped smoking for illness were included in the current smokers category). The test for interaction was performed by using a likelihood ratio test comparing models with and without cross-product term.

All statistical analyses were carried out using Stata version 15.0 (Stata Corp, TX, USA). R version 3.5.2 was used to graph results. Statistical significance was set at two-tailed *P* < 0.05.

## Results

Of the 440,763 participants included, the mean baseline age was 50.9 (± 10.3) years, 60.2% were women, and 43.7% were from urban areas. The mean BMI was 23.7 (± 3.3) kg/m^2^, with 3.8% being underweight and 4.0% being obese. Obese participants were more likely to be women, live in urban areas, and have diabetes (Table 1). In contrast, underweight participants were more likely to live in rural areas, be current smokers, and report poor self-rated health.

**Table 1:** Baseline characteristics of the study participants according to BMI in the primary analysis

	< 18.5	18.5 to < 20.0	20.0 to < 22.5	22.5 to < 25.0	25.0 to < 27.5	27.5 to < 30.0	≥ 30.0	All
Number of participants, *n* (%)	16,701 (3.8)	36,957 (8.4)	114,513 (26.0)	126,914 (28.8)	88,193 (20.0)	39,922 (9.1)	17,563 (4.0)	440,763
BMI, kg/m^2^	17.6	19.3	21.3	23.7	26.1	28.5	31.8	23.7
**Socio-demographic characteristics**								
Age, years	53.4	51.0	50.2	50.6	51.1	51.4	51.6	50.9
Women, %	61.2	55.9	58.7	60.8	60.0	62.1	70.2	60.2
Urban area, %	32.4	32.0	37.0	44.6	50.7	54.1	56.5	43.7
Middle school and higher, %	49.0	50.0	50.7	51.7	51.1	49.4	47.2	50.7
**Dietary and lifestyle factors**								
Male current smoker*, %	77.2	75.7	71.3	65.7	62.6	61.5	61.5	67.4
Female current smoker*, %	4.3	3.0	2.4	2.2	2.0	2.0	2.3	2.3
Male daily drinker, %	19.8	21.8	22.6	21.4	19.6	18.7	17.0	20.9
Female daily drinker, %	1.1	0.9	1.0	0.9	0.9	0.8	0.7	0.9
Physical activity, MET h/d	21.8	22.4	22.6	22.1	21.4	20.7	19.9	21.9
Regular consumption of**								
Fresh fruits, %	22.7	25.7	27.5	28.7	29.3	29.4	29.1	28.2
Fresh vegetables, %	97.9	98.1	98.2	98.3	98.5	98.4	98.2	98.3
Red meat, %	43.4	44.9	46.6	48.5	49.8	50.3	50.0	48.0
**Anthropometric measures**								
Male WC, cm	67.6	71.2	76.1	82.8	89.0	94.8	101.8	82.2
Female WC, cm	64.0	68.3	73.1	78.8	84.3	89.6	96.5	78.9
**Medical history and health status**								
Diabetes, %	2.4	2.9	3.7	5.1	6.6	7.7	9.7	5.2
Self-rated poor health, %	13.4	10.0	8.4	7.3	7.5	8.4	10.3	8.3

Baseline characteristics were adjusted for age, sex, and study area as appropriate

*BMI* body-mass index, *MET h/d* metabolic equivalent of task hours per day, *WC* waist circumference

*Included former smokers who had stopped smoking due to illness

**Reported consuming at least 4–6 days per week

During a median follow-up of 10.0 years (4,467,205 person-years), 30,237 deaths were recorded, including 2346 sepsis-related deaths and 27,891 non-sepsis-related deaths. After excluding the first 3 years of follow-up (leaving a median of 7.1 years and 3,134,870 person-years for the analysis), the number of deaths was 24,522, including 1957 sepsis-related deaths and 22,565 non-sepsis-related deaths. Respiratory diseases were the dominating underlying causes of sepsis-related deaths (Additional file 1: Table S2). The survival curves by BMI showed that the group of BMI < 18.5 kg/m^2^ had the lowest survival probabilities (Additional file 1: Figure S2).

Table 2 shows moderate changes of the HRs, especially for underweight and obese participants, with progressively stricter exclusions. In the primary analysis, compared with reference BMI group of 22.5 to < 25.0 kg/m^2^, the multivariable-adjusted HRs (95% CIs) for sepsis-related mortality were 2.42 (2.07–2.84), 1.59 (1.36–1.85), 1.21 (1.06–1.38), 0.97 (0.83–1.13), 0.98 (0.80–1.21), and 1.22 (0.93–1.60) for BMI of < 18.5, 18.5 to < 20.0, 20.0 to < 22.5, 25.0 to < 27.5, 27.5 to < 30.0, and ≥ 30.0 kg/m^2^. For BMI < 25 kg/m^2^, the risk of sepsis-related mortality increased with reduced BMI; the HR (95% CI) for per 1 kg/m^2^ lower BMI was 1.15 (1.13–1.18).

**Table 2:** Adjusted HRs for sepsis-related mortality by baseline BMI, applying various exclusions

	Baseline BMI (kg/m^**2**^)						
	< 18.5	18.5 to < 20.0	20.0 to < 22.5	22.5 to < 25.0	25.0 to < 27.5	27.5 to < 30.0	≥ 30.0
**Multivariable model with no exclusions, adjusted for smoking status**							
Participants/deaths	21,916/911	44,188/757	131,839/1133	145,408/850	101,523/492	46,423/262	20,964/125
Deaths/PYs* (/1000)	2.61	1.42	0.92	0.69	0.61	0.76	0.85
HRs (95% CIs)	3.57 (3.24–3.94)	2.00 (1.81–2.21)	1.33 (1.22–1.46)	1.00	0.88 (0.78–0.98)	1.07 (0.93–1.23)	1.17 (0.96–1.41)
**Participants without known chronic diseases at baseline, adjusted for smoking status**							
Participants/deaths	17,260/368	37,716/346	116,084/613	128,367/506	89,129/302	40,352/140	17,775/71
Deaths/PYs* (/1000)	1.30	0.74	0.55	0.46	0.42	0.46	0.56
HRs (95% CIs)	2.60 (2.26–2.99)	1.56 (1.36–1.80)	1.19 (1.06–1.34)	1.00	0.92 (0.80–1.06)	0.99 (0.81–1.19)	1.17 (0.91–1.51)
**Participants without known chronic diseases at baseline, adjusted for smoking status, and excluding the first 3 years of follow-up (primary prespecified analysis)**							
Participants/deaths	16,701/280	36,957/288	114,513/513	126,914/425	88,193/271	39,922/118	17,563/62
Deaths/PYs* (/1000)	1.44	0.90	0.66	0.55	0.53	0.54	0.69
HRs (95% CIs)	2.42 (2.07–2.84)	1.59 (1.36–1.85)	1.21 (1.06–1.38)	1.00	0.97 (0.83–1.13)	0.98 (0.80–1.21)	1.22 (0.93–1.60)
**Never smokers without known chronic diseases at baseline, excluding the first 3 years of follow-up**							
Participants/deaths	10,354/93	21,524/109	70,419/219	81,426/194	56,428/137	26,190/68	12,597/42
Deaths/PYs* (/1000)	0.80	0.63	0.47	0.38	0.39	0.41	0.55
HRs (95% CIs)	2.02 (1.57–2.60)	1.61 (1.27–2.04)	1.26 (1.04–1.53)	1.00	1.02 (0.82–1.28)	1.07 (0.81–1.41)	1.39 (0.99–1.95)

Multivariable model was stratified by age, sex, and study area. Covariates adjusted for in the model included education (no formal school, primary school, middle school, high school, college, or university or higher); alcohol consumption (non-weekly drinker, former weekly drinker, weekly drinker, daily drinker: < 30 g/day or ≥ 30 g/day of pure alcohol); physical activity (MET h/d); intake frequency of fresh fruits, vegetables, and red meat (days/week: calculated by assigning participants to the midpoint of their consumption category); and smoking status (never smoker, former smoker who had stopped for reasons other than illness, current smoker or former smoker who had stopped due to illness: 1–9, 10–19, 20–29, or ≥ 30 cigarettes or equivalent per day; not included in the last model)

*HR* hazard ratio, *CI* confidence interval, *BMI* body-mass index, *PYs* person-years, *MET h/d* metabolic equivalent of task hours per day

^*^Adjusted for age, sex, and study area

**Fig. 1:**
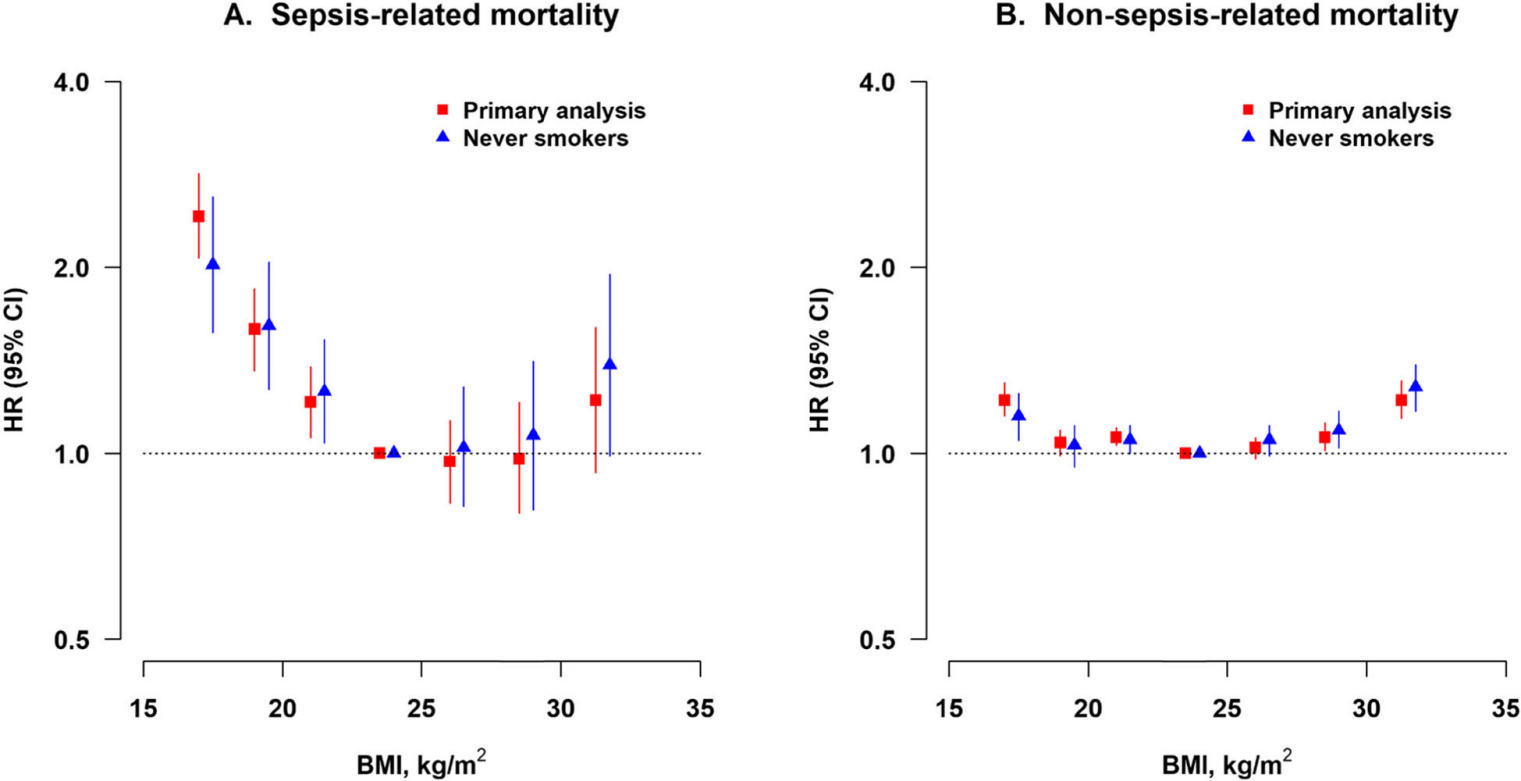
Association between BMI and sepsis-related and non-sepsis-related mortality. HR, hazard ratio; CI, confidence interval; BMI, body-mass index. Primary analysis (solid squares; *n* = 440,763): participants without known chronic diseases at baseline and excluding the first 3 years of follow-up. Never smokers (solid triangles; *n* = 278,938): further excluding ever smokers. Multivariable model was stratified by age, sex, and study area and adjusted for the same set of covariates as in Table 2

When the analysis was further restricted to the never smokers, the HR for underweight decreased slightly (2.02; 95% CI 1.57–2.60) and that for obesity increased (1.39; 95% CI 0.99–1.95) (Table 2). The association of BMI with sepsis-related mortality did not change substantially for most of the sensitivity analyses (Additional file 1: Table S3, Figure S3). However, the HRs for both the underweight (1.74; 95% CI 1.48–2.03) and low-normal weight (1.42; 95% CI 1.21–1.65) groups were moderately attenuated after adjustment for the incidence of COPD during follow-up. When comparing the associations of two outcomes, that is, sepsis-related and non-sepsis-related mortality, with the BMI, underweight and low- and mid-normal weight had a more significant influence on the risk of sepsis-related mortality (Fig. 1, Table 2, and Additional file 1: Table S4).

**Fig. 2:**
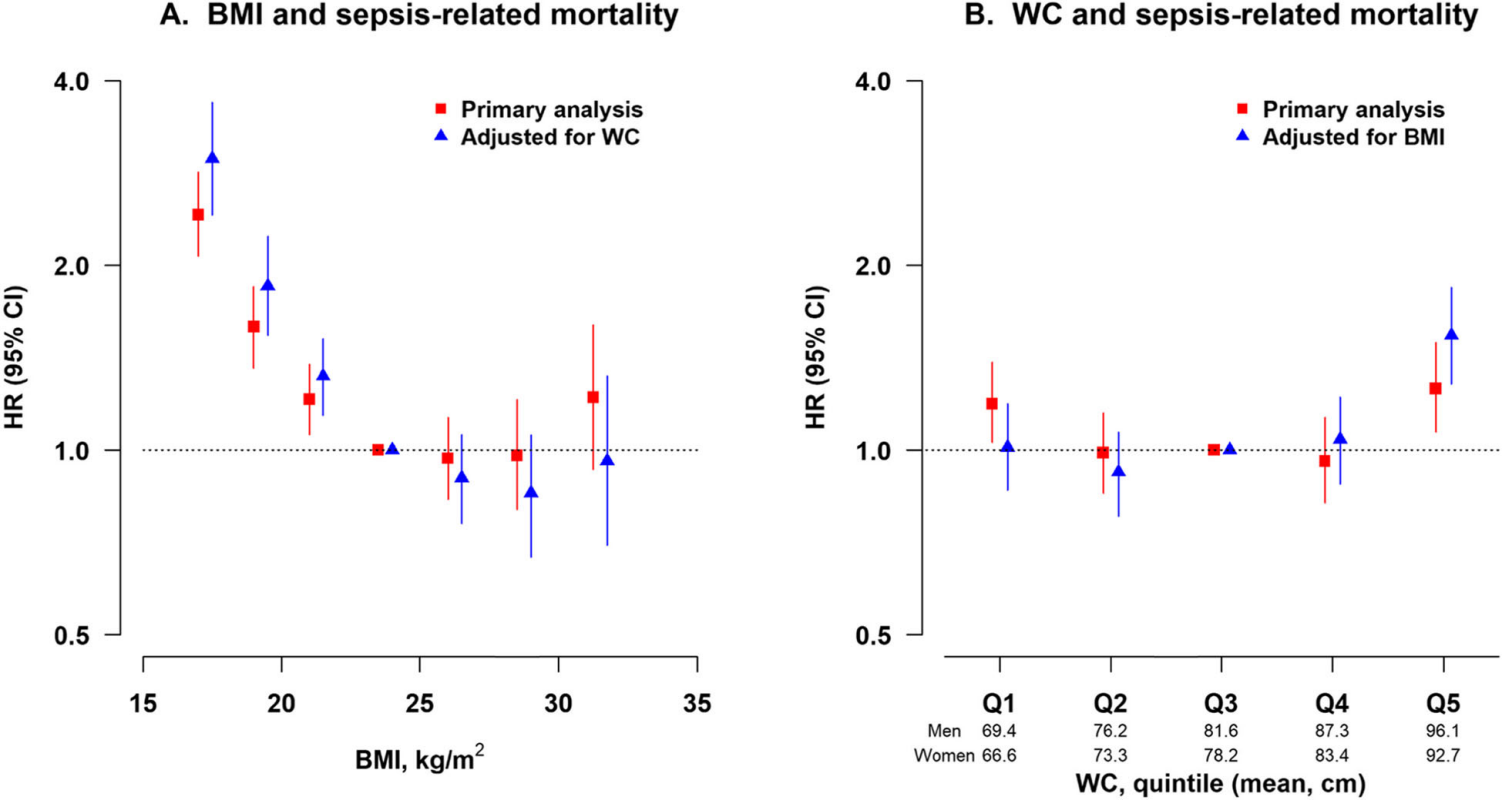
Association of sepsis-related mortality with BMI or WC. HR, hazard ratio; CI, confidence interval; BMI, body-mass index; WC, waist circumference. Primary analysis (solid squares; *n* = 440,763): participants without known chronic diseases at baseline and excluding the first 3 years of follow-up. Multivariable model was stratified by age, sex, and study area and adjusted for the same set of covariates as in Table 2. In the analysis of WC and sepsis-related mortality, we additionally adjusted for hip circumference. Further mutual adjustment (solid triangles; *n* = 440,763)

In the primary analysis of BMI and sepsis-related mortality, further adjustment for WC led to slightly augmentation of the effect size for the lower BMI groups and null association in the obese group (Fig. 2, Additional file 1: Table S5). The similar analysis was conducted for the association between WC and sepsis-related mortality. Compared with the middle quintile group (Q3), only the highest quintile group (Q5) showed an increased risk of sepsis-related mortality after further adjustment for BMI (HR = 1.54; 95% CI 1.28–1.84) (Fig. 2, Additional file 1: Table S6).

**Fig. 3:**
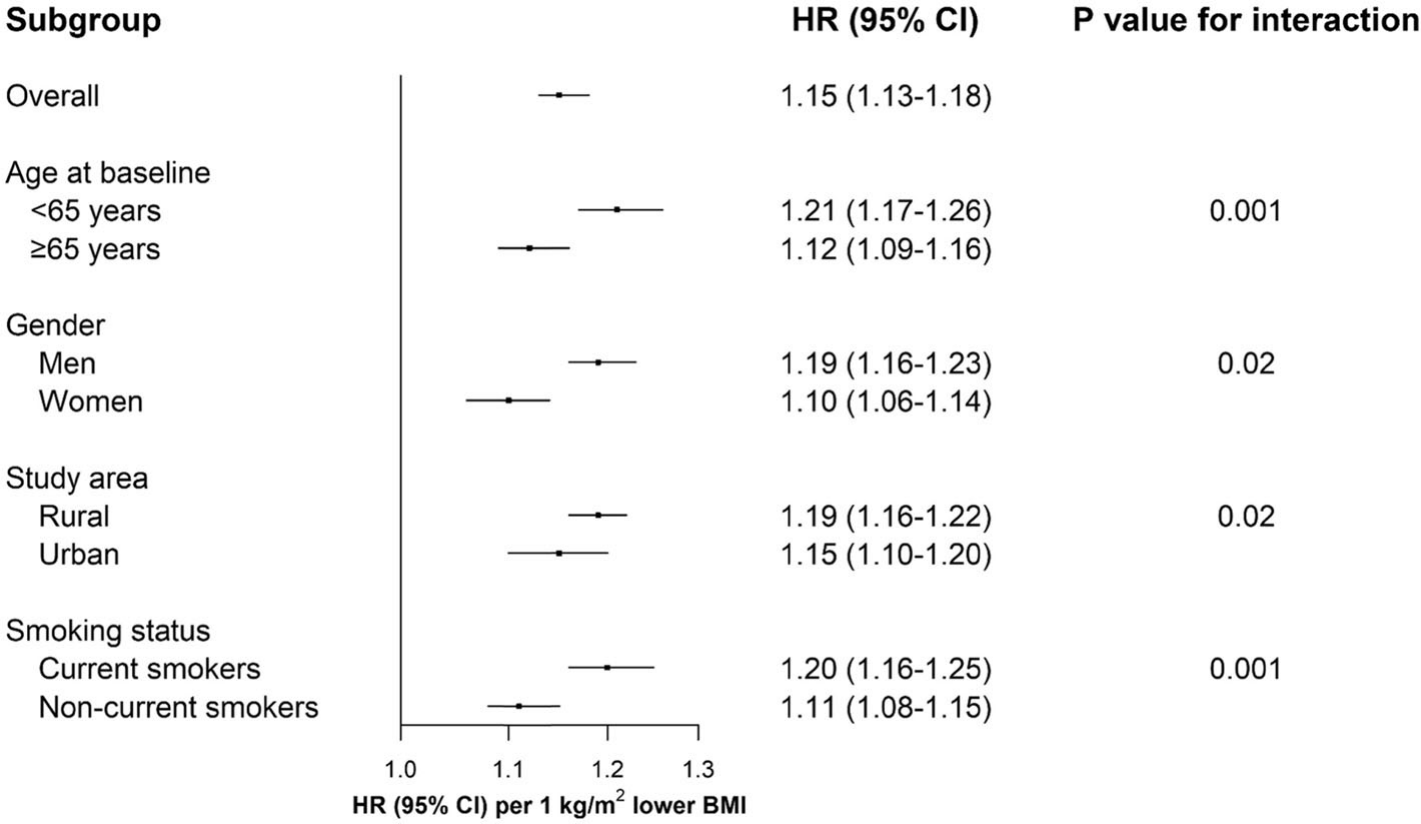
Subgroup analysis of adjusted HRs per 1 kg/m^2^ lower BMI for sepsis-related mortality at BMI < 25 kg/m^2^. HR, hazard ratio; CI, confidence interval; BMI, body-mass index. Primary analysis was conducted among participants without known chronic diseases at baseline and excluded the first 3 years of follow-up. Multivariable model was stratified by age, sex, and study area as appropriate and adjusted for the same set of covariates as in Table 2

In the subgroup analyses, the more remarkable differences across prespecified baseline factors were observed in the lower BMI groups (Additional file 1: Figure S4). The adjusted HRs per 1 kg/m^2^ lower BMI for sepsis-related mortality at BMI < 25 kg/m^2^ were stronger in participants aged less than 65 years, men, rural residents, or current smokers than their counterparts, respectively (all *P* for interaction < 0.05, Fig. 3).

## Discussion

In this large prospective Chinese cohort study, both BMI below the range of 22.5 to < 25.0 kg/m^2^ and abdominal obesity were associated with a higher long-term risk of sepsis-related mortality. The changes in the effect size after mutual adjustment for BMI and WC suggested the importance of abdominal obesity as an independent risk factor for the burden of sepsis.

Few previous studies in HICs have addressed the association between BMI and long-term risk of sepsis-related mortality, with seemingly inconsistent findings. In an analysis of US National Walkers’ and Runners’ Health Study of 101,351 runners and 33,411 walkers, which were followed up for an average of 11.6 years, abdominally obese (WC > 102 cm in men and > 88 cm in women) was associated with an increased risk of mortality with sepsis as underlying or contributing cause (HR = 1.74; 95% CI 1.03–2.87). However, BMI was not associated with sepsis mortality after adjustment for WC [17]. Another study using data from the Reasons for Geographic and Racial Differences in Stroke (REGARDS) study examined the association between obesity and subsequent first sepsis episode and observed similar results [26]. Findings from the 15-year follow-up of 64,027 Norwegians in the HUNT Study showed that the adjusted HRs (95% CIs) for mortality from bloodstream infection were 1.09 (0.81–1.47) at BMI 25.0–29.9 kg/m^2^, 1.49 (1.02–2.17) at BMI 30.0–34.9 kg/m^2^, 2.56 (1.45–4.53) at BMI 35.0–39.9 kg/m^2^, and 5.67 (2.56–12.58) at BMI ≥ 40.0 kg/m^2^, compared with normal weight of BMI 18.5–24.9 kg/m^2^. Underweight of BMI < 18.5 kg/m^2^ was not associated with bloodstream infection mortality (HR = 0.96; 95% CI 0.13–6.94) [16]. In contrast, the Japan Collaborative Cohort Study for Evaluation of Cancer Risk (JACC Study) of 110,792 Japanese adults, followed up from 1988 to 2003, reported that the risk for pneumonia death was higher in underweight participants with BMI 10.0–17.9 kg/m^2^ (HR = 2.1; 95% CI 1.7–2.5), and lower in those with BMI 23.0–24.9 kg/m^2^ (0.7; 0.6–0.9) and 25.0–32.9 kg/m^2^ (0.6; 0.5–0.8), than those with BMI 18.0–22.9 kg/m^2^ [15].

Intriguingly, the findings of the present study, conducted in a Chinese population facing a double burden of malnutrition, add to the available evidence that the association between the continuum of BMI and sepsis-related mortality was reverse J-shaped. On the one hand, obesity, or rather, abdominal obesity measured by WC, was associated with an increased future risk of sepsis-related mortality. This result is similar with previous studies conducted in the Western population [16, 17, 26]. Animal models of sepsis have replicated similar findings that obese animals had increased mortality, a greater number of complications, and an altered systemic inflammatory response as compared to lean animals [27]. We speculate that such connection might be the result of visceral fat which expresses more proinflammatory cytokines, leading to a chronic inflammatory state and an altered immune response to infection [26].

On the contrary, CKB participants who were underweight or low- and mid-normal weight were at greater risk of sepsis-related mortality than those with high-normal weight, with the risk increasing with decreasing BMI. Similar findings were reported in the JACC study [15], but the null association was observed in studies of the Western population [16, 26], most possibly due to the underrepresentation of underweight participants and one combined group of BMI 18.5–24.9 kg/m^2^ in their analyses. Less nutritional reserves and altered immune response may be a possible mechanism behind the association between lower BMI and increased risk of sepsis-related mortality [28]. Besides, in the CKB population, nearly half of the sepsis-related deaths were presented with COPD as the underlying cause of death; also, the HRs for the underweight and low-normal weight groups were moderately attenuated after adjustment for the incidence of COPD during follow-up. These may suggest that COPD at least partly mediates the process from lower BMI to sepsis-related mortality.

To the best of our knowledge, this large prospective study of Chinese adults is the first to assess the associations between anthropometric measures and future risk of sepsis-related mortality in a population outside HICs, including both urban and rural areas and facing the dual burden of underweight and obesity. The large sample size enabled us to further subdivide the BMI categories in the normal range and found that even participants with low- and mid-normal weight had increased sepsis-related mortality risk. Both BMI and WC were measured using standardized examination procedures and calibrated equipment, allowing us to better phenotype body composition. Our primary analyses excluded participants known to have major chronic diseases at baseline and omitted the first 3 years of follow-up to avoid reverse causation. More sensitivity analyses were performed to detect the presence of any biases, including restriction of analysis to never smokers to avoid residual confounding by smoking.

Our study has limitations worth mentioning. First of all, the algorithm to identify sepsis-related death was based on ICD codes for acute infections. However, this approach warrants relatively accurate identification of sepsis, according to our previous study [1] and validation in the present study. It might be the only pragmatic approach to investigate sepsis-related mortality in LMICs with limited electronic medical records. Also, misclassification of sepsis-related mortality should be non-differential on BMI categories. Second, BMI and WC of all CKB participants were measured only once at baseline, but they were suggested to have high levels of concordance in replicate measures taken from the same adults some years apart [12]. Third, being an observational study, residual confounding and inferring causality are often challenging.

## Conclusion

In summary, this population-based, prospective cohort of Chinese adults provides convincing evidence that underweight, lower normal weight, and abdominal obesity are associated with increased future risk of sepsis-related mortality over 10 years. The double burden of malnutrition, together with a higher probability of infection and organ dysfunction, and higher case-fatality rates [29], indicates a heavy sepsis burden faced by LMICs. To make long-term public health gains, a particular focus should be on modifiable risk factors for the primary prevention of sepsis.

## Supplementary information

**Additional file 1: Text S1.** Members of the China Kadoorie Biobank collaborative group. **Table S1.** ICD-10 codes related to sepsis-related mortality. **Table S2.** Number of major underlying cause-specific deaths by sepsis-related and non-sepsis-related mortality. **Table S3.** Sensitivity analysis for BMI and sepsis-related mortality by applying additional adjustments or exclusions. **Table S4.** Adjusted HRs for non-sepsis-related mortality by baseline BMI, applying various exclusions. **Table S5.** Adjusted HRs (95% CIs) for association between BMI and sepsis-related mortality. **Table S6.** Adjusted HRs (95% CIs) for association between WC and sepsis-related mortality. **Figure S1.** Flow diagram for study participants in the primary analysis. **Figure S2.** Kaplan Meier survival probabilities by BMI. **Figure S3.** Adjusted HRs per 1 kg/ m^2^ lower BMI for sepsis-related mortality at BMI <25 kg/m^2^ by applying additional adjustments or exclusions. **Figure S4.** Association between BMI and sepsis-related mortality by baseline factors.

## Data Availability

The access policy and procedures are available at www.ckbiobank.org.

## Abbreviations

BMI: Body-mass index
WC: Waist circumference
HR: Hazard ratio
CI: Confidence interval
HICs: High-income countries
LMICs: Low- and middle-income countries
CKB: China Kadoorie Biobank
HC: Hip circumference
DSPs: Disease surveillance points
ICD-10: International Statistical Classification of Diseases and Related Health Problems 10th Revision
COPD: Chronic obstructive pulmonary disease
