# Supplementary material for: Body-mass index and long-term risk of sepsis-related mortality: a population-based cohort study of 0.5 million Chinese adults
Source: Crit Care. 2020 Aug 31;24:534. doi: 10.1186/s13054-020-03229-2 (PMC7457784; doi:10.1186/s13054-020-03229-2)
Supplement: Supplementary file 1 — Additional file 1: Text S1. Members of the China Kadoorie Biobank collaborative group. Table S1. ICD-10 codes related to sepsis-related mortality. Table S2. Number of major underlying cause-specific deaths by sepsis-related and non-sepsis-related mortality. Table S3. Sensitivity analysis for BMI and sepsis-related mortality by applying additional adjustments or exclusions. Table S4. Adjusted HRs for non-sepsis-related mortality by baseline BMI, applying various exclusions. Table S5. Adjusted HRs (95% CIs) for association between BMI and sepsis-related mortality. Table S6. Adjusted HRs (95% CIs) for association between WC and sepsis-related mortality. Figure S1. Flow diagram for study participants in the primary analysis. Figure S2. Kaplan Meier survival probabilities by BMI. Figure S3. Adjusted HRs per 1 kg/ m2 lower BMI for sepsis-related mortality at BMI <25 kg/m2 by applying additional adjustments or exclusions. Figure S4. Association between BMI and sepsis-related mortality by baseline factors. [file 13054_2020_3229_MOESM1_ESM.docx]

**Text S1. Members of the China Kadoorie Biobank collaborative group**

**International Steering Committee:** Junshi Chen, Zhengming Chen (PI), Robert Clarke, Rory Collins, Yu Guo, Liming Li (PI), Jun Lv, Richard Peto, Robin Walters. **International Co-ordinating Centre, Oxford:** Daniel Avery, Ruth Boxall, Derrick Bennett, Yumei Chang, Yiping Chen, Zhengming Chen, Robert Clarke, Huaidong Du, Simon Gilbert, Alex Hacker, Mike Hill, Michael Holmes, Andri Iona, Christiana Kartsonaki, Rene Kerosi, Ling Kong, Om Kurmi, Garry Lancaster, Sarah Lewington, Kuang Lin, John McDonnell, Iona Millwood, Qunhua Nie, Jayakrishnan Radhakrishnan, Paul Ryder, Sam Sansome, Dan Schmidt, Paul Sherliker, Rajani Sohoni, Becky Stevens, Iain Turnbull, Robin Walters, Jenny Wang, Lin Wang, Neil Wright, Ling Yang, Xiaoming Yang. **National Co-ordinating Centre, Beijing:** Zheng Bian, Yu Guo, Xiao Han, Can Hou, Jun Lv, Pei Pei, Chao Liu, Yunlong Tan, Canqing Yu. **10 Regional Co-ordinating Centres: Qingdao CDC:** Zengchang Pang, Ruqin Gao, Shanpeng Li, Shaojie Wang, Yongmei Liu, Ranran Du, Yajing Zang, Liang Cheng, Xiaocao Tian, Hua Zhang, Yaoming Zhai, Feng Ning, Xiaohui Sun, Feifei Li. **Licang CDC:** Silu Lv, Junzheng Wang, Wei Hou. **Heilongjiang Provincial CDC:** Mingyuan Zeng, Ge Jiang, Xue Zhou. **Nangang CDC:** Liqiu Yang, Hui He, Bo Yu, Yanjie Li, Qinai Xu,Quan Kang, Ziyan Guo. **Hainan Provincial CDC:** Dan Wang, Ximin Hu, Jinyan Chen, Yan Fu, Zhenwang Fu, Xiaohuan Wang. **Meilan CDC:** Min Weng, Zhendong Guo, Shukuan Wu,Yilei Li, Huimei Li, Zhifang Fu. **Jiangsu Provincial CDC:** Ming Wu, Yonglin Zhou, Jinyi Zhou, Ran Tao, Jie Yang, Jian Su. **Suzhou CDC:** Fang liu, Jun Zhang, Yihe Hu, Yan Lu, , Liangcai Ma, Aiyu Tang, Shuo Zhang, Jianrong Jin, Jingchao Liu. **Guangxi Provincial CDC:** Zhenzhu Tang, Naying Chen, Ying Huang. **Liuzhou CDC:** Mingqiang Li, Jinhuai Meng, Rong Pan, Qilian Jiang, Jian Lan,Yun Liu, Liuping Wei, Liyuan Zhou, Ningyu Chen Ping Wang, Fanwen Meng, Yulu Qin,, Sisi Wang. **Sichuan Provincial CDC:** Xianping Wu, Ningmei Zhang, Xiaofang Chen,Weiwei Zhou. **Pengzhou CDC:** Guojin Luo, Jianguo Li, Xiaofang Chen, Xunfu Zhong, Jiaqiu Liu, Qiang Sun. **Gansu Provincial CDC:** Pengfei Ge, Xiaolan Ren, Caixia Dong. **Maiji CDC:** Hui Zhang, Enke Mao, Xiaoping Wang, Tao Wang, Xi zhang. **Henan Provincial CDC:** Ding Zhang, Gang Zhou, Shixian Feng, Liang Chang, Lei Fan. **Huixian CDC:** Yulian Gao, Tianyou He, Huarong Sun, Pan He, Chen Hu, Xukui Zhang, Huifang Wu, Pan He. **Zhejiang Provincial CDC:** Min Yu, Ruying Hu, Hao Wang. Tongxiang CDC: Yijian Qian, Chunmei Wang, Kaixu Xie, Lingli Chen, Yidan Zhang, Dongxia Pan, Qijun Gu. **Hunan Provincial CDC:** Yuelong Huang, Biyun Chen, Li Yin, Huilin Liu, Zhongxi Fu, Qiaohua Xu. **Liuyang CDC:** Xin Xu, Hao Zhang, Huajun Long, Xianzhi Li, Libo Zhang, Zhe Qiu.

**Table S1. ICD-10 codes related to sepsis-related mortality**

| **ICD title** | **ICD-10 codes** |
| --- | --- |
| I Certain infectious and parasitic diseases | A00-A09, A15-A16, A19-A28, A31-A43, A46, A48-A51, A53-A56, A60, A69, A71, A74-A75, A79, A81-A89, A92-A94, A96, A98-A99, B00-B02, B05-B06, B08-B09, B20, B30, B25-B27, B33-B35, B37-B46, B48-B59, B65-B69, B74, B76-B77, B88-B89, B95-B99 |
| III Diseases of the blood and blood-forming organs and certain disorders involving the immune mechanism | D73.3, D76.2, D77 |
| IV Endocrine, nutritional and metabolic diseases | E06.0, E23.6, E27.8, E32.1 |
| VI Diseases of the nervous system | G00-G02, G04-G08, G21.3, G22, G53, G61, G63.0, G72.4, G73.4, G94.0 |
| VII Diseases of the eye and adnexa | H00.0, H01.0, H03, H04.0, H04.3, H05.0, H06.1, H10, H13.0, H13.1, H15.0, H15.1, H16, H19.0, H19.1, H19.2, H20, H22.0, H30, H32.0, H42.8, H44.0, H44.1, H45.1, H48.0, H48.1, H58.8 |
| IX Diseases of the circulatory system | I00, I01, I02, I30.1, I32.0, I32.1, I33.0, I38, I39, I40.0, I41.0, I41.1, I41.2, I43.0, I52.0, I52.1, I68.1, I79.0, I79.1, I80.0, I80.2, I80.3, I98.0, I98.1 |
| X Diseases of the respiratory system | J00-J06, J09-J18, J20-J22, J31, J32, J34.0, J36, J37, J38.3, J38.7, J39.0, J39.1, J40, J41, J42, J44.0, J44.1, J69, J85-J86, J95.0 |
| XI Diseases of the digestive system | K04.0, K04.1, K04.4, K04.5, K04.6, K04.7, K05.0, K05.1, K05.2, K05.3, K10.2, K10.3, K11.2, K11.3, K11.4, K12, K13.0, K14.0, K20, K22.3, K23.0, K25.1, K25.2, K25.5, K26.1, K26.2, K26.5, K26.6, K27.1, K27.2, K27.5, K27.6, K28.1, K28.2, K28.5, K31.6, K35, K36, K37, K38.3, K40.1, K40.4, K41.1, K41.4, K42.1, K43.1, K43.4, K44.1, K45.1, K46.1, K55.0, K57.0, K57.2, K57.4, K57.8, K60.3, K60.4, K60.5, K61, K63.0, K63.1, K63.2, K65.0, K65.9, K67, K75.0, K75.1, K77.0, K80.0, K80.1, K80.3, K80.4, K81, K82.2, K82.3, K83.0, K83.2, K83.3, K85, K87 |
| XII Diseases of the skin and subcutaneous tissue | L00-L08 |
| XIII Diseases of the musculoskeletal system and connective tissue | M00-M03, M46.2, M46.3, M46.5, M49.0, M49.1, M49.2, M49.3, M60.0, M63.0, M63.1, M63.2, M65.0, M65.1, M68.0, M71.0, M71.1, M72.6, M72.8, M73.0, M73.1, M86, M90.0, M90.1, M90.2 |
| XIV Diseases of the genitourinary system | N08.0, N13.6, N15.1, N15.9, N16.0, N22.0, N29.0, N29.1, N30.0, N30.3, N30.8, N32.1, N32.3, N33, N34.0, N34.2, N36.0, N37.0, N39.0, N41.0, N41.2, N41.3, N43.1, N45, N48.1, N48.2, N49, N51, N61, N70-N77 |
| XV Pregnancy, childbirth and the puerperium | O03.0, O03.5, O04.0, O04.5, O05.0, O05.5, O06.0, O06.5, O07.0, O07.5, O08.0, O23, O29.0, O41.1, O74.0, O75.3, O85, O86, O89.0, O91, O98 |
| XVI Certain conditions originating in the perinatal period | P23, P24, P35-P39, P77, P78.0, P78.1 |
| XVIII Symptoms, signs and abnormal clinical and laboratory findings, not elsewhere classified | R02, R09.1, R10.0, R57.8, R65.0, R65.1 |
| XIX Injury, poisoning and certain other consequences of external causes | S36.3, S36.4, S36.5, S36.6, T80.2, T81.4, T82.6, T82.7, T83.5, T83.6, T84.5, T84.6, T84.7, T85.7, T87.4, T88.0 |

**Table S2. Number of major underlying cause-specific deaths by sepsis-related and non-sepsis-related mortality**

| **Cause-specific deaths** | **ICD-10 codes** | **Sepsis-related mortality** | **Non-sepsis-related mortality** |
| --- | --- | --- | --- |
| Number of all deaths |  | 1957 | 22565 |
| Ischaemic heart disease | I20-I25 | 37 | 3536 |
| Stroke | I60-I61, I63-I64 | 62 | 4250 |
| Haemorrhagic stroke | I61 | 23 | 2880 |
| Ischaemic stroke | I63 | 32 | 1134 |
| Cancer | C00-C97 | 202 | 8283 |
| Diseases of the respiratory system | J00-J99 | 1181 | 268 |
| Chronic obstructive pulmonary disease | J41-J44 | 830 | 162 |
| Pneumonia | J12-J18 | 305 | 0 |
| Other respiratory diseases | J00-J99 except for J12-J18, J41-J44 | 46 | 106 |
| Infectious and parasitic diseases | A00-B99 | 101 | 207 |

**Table S3. Sensitivity analysis for BMI and sepsis-related mortality by applying additional adjustments or exclusions**

| **Applying additional adjustments or exclusions** | **Baseline BMI (kg/m^2^)** | | | | | | |
| --- | --- | --- | --- | --- | --- | --- | --- |
|  | **<18.5** | **18.5 to <20.0** | **20.0 to <22.5** | **22.5 to <25.0** | **25.0 to <27.5** | **27.5 to <30.0** | **≥30.0** |
| Primary prespecified analysis^a^ | | | | | | | |
| HRs (95% CIs) | 2.42 (2.07-2.84) | 1.59 (1.36-1.85) | 1.21 (1.06-1.38) | 1.00 | 0.97 (0.83-1.13) | 0.98 (0.80-1.21) | 1.22 (0.93-1.60) |
| + adjusted for baseline household income, occupation, and marital status | | | | | | | |
| HRs (95% CIs) | 2.36 (2.02-2.77) | 1.56 (1.34-1.82) | 1.20 (1.06-1.37) | 1.00 | 0.98 (0.84-1.14) | 0.99 (0.80-1.21) | 1.22 (0.93-1.60) |
| + adjusted for incident COPD during follow-up | | | | | | | |
| HRs (95% CIs) | 1.74 (1.48-2.03) | 1.42 (1.21-1.65) | 1.17 (1.03-1.34) | 1.00 | 0.97 (0.83-1.13) | 0.95 (0.78-1.17) | 1.18 (0.90-1.56) |
| + adjusted for incident pneumonia during follow-up | | | | | | | |
| HRs (95% CIs) | 2.27 (1.94-2.65) | 1.55 (1.33-1.81) | 1.21 (1.06-1.38) | 1.00 | 1.00 (0.86-1.17) | 0.98 (0.79-1.20) | 1.14 (0.87-1.50) |
| + adjusted for other incident respiratory diseases during follow-up | | | | | | | |
| HRs (95% CIs) | 2.32 (1.98-2.71) | 1.55 (1.33-1.80) | 1.20 (1.05-1.36) | 1.00 | 0.97 (0.83-1.13) | 0.98 (0.79-1.20) | 1.18 (0.90-1.55) |
| + adjusted for incident TB during follow-up | | | | | | | |
| HRs (95% CIs) | 2.33 (1.99-2.72) | 1.56 (1.34-1.82) | 1.20 (1.06-1.37) | 1.00 | 0.99 (0.85-1.15) | 0.99 (0.81-1.22) | 1.24 (0.94-1.62) |
| + adjusted for incident cancer during follow-up | | | | | | | |
| HRs (95% CIs) | 2.45 (2.09-2.86) | 1.60 (1.37-1.86) | 1.21 (1.06-1.37) | 1.00 | 0.97 (0.84-1.13) | 0.97 (0.79-1.19) | 1.22 (0.93-1.60) |
| + adjusted for incident diabetes during follow-up | | | | | | | |
| HRs (95% CIs) | 2.49 (2.13-2.92) | 1.62 (1.39-1.89) | 1.23 (1.08-1.40) | 1.00 | 0.96 (0.82-1.12) | 0.95 (0.78-1.17) | 1.17 (0.89-1.54) |
| + excluded diabetes at baseline | | | | | | | |
| HRs (95% CIs) | 2.53 (2.15-2.99) | 1.63 (1.39-1.92) | 1.26 (1.10-1.45) | 1.00 | 0.98 (0.83-1.16) | 1.00 (0.80-1.26) | 1.25 (0.92-1.69) |
| + excluded self-rated poor health status at baseline | | | | | | | |
| HRs (95% CIs) | 2.50 (2.11-2.97) | 1.62 (1.37-1.91) | 1.27 (1.10-1.46) | 1.00 | 0.99 (0.83-1.16) | 1.00 (0.80-1.25) | 1.30 (0.97-1.74) |

BMI = body-mass index; HR = hazard ratio; CI = confidence interval; COPD = chronic obstructive pulmonary disease; TB = tuberculosis.

^a^Primary analysis was conducted among participants without known chronic diseases at baseline and excluded the first 3 years of follow-up. Multivariable model was stratified by age, sex, and study area and adjusted for the same set of covariates as in the Table 2.

**Table S4. Adjusted HRs for non-sepsis-related mortality by baseline BMI, applying various exclusions**

| **Applying various exclusions** | **Baseline BMI (kg/m^2^)** | | | | | | |
| --- | --- | --- | --- | --- | --- | --- | --- |
|  | **<18.5** | **18.5 to <20.0** | **20.0 to <22.5** | **22.5 to <25.0** | **25.0 to <27.5** | **27.5 to <30.0** | **≥30.0** |
| Multivariable model with no exclusions, adjusted for smoking status | | | | | | | |
| Participants/deaths | 21916/3172 | 44188/4246 | 131839/10214 | 145408/9800 | 101523/6924 | 46423/3272 | 20964/1691 |
| Deaths/PYs^a^ (/1000) | 11.18 | 8.84 | 8.31 | 7.59 | 7.69 | 8.04 | 9.32 |
| HRs (95% CIs) | 1.41 (1.35-1.47) | 1.14 (1.09-1.18) | 1.09 (1.06-1.12) | 1.00 | 1.02 (0.99-1.05) | 1.05 (1.01-1.09) | 1.20 (1.14-1.27) |
| Participants without known chronic diseases at baseline, adjusted for smoking status | | | | | | | |
| Participants/deaths | 17260/1908 | 37716/2908 | 116084/7429 | 128367/7177 | 89129/4972 | 40352/2328 | 17775/1169 |
| Deaths/PYs^a^ (/1000) | 8.51 | 7.00 | 6.69 | 6.14 | 6.15 | 6.44 | 7.52 |
| HRs (95% CIs) | 1.31 (1.25-1.38) | 1.10 (1.06-1.15) | 1.07 (1.04-1.11) | 1.00 | 1.01 (0.97-1.05) | 1.05 (1.00-1.10) | 1.20 (1.13-1.28) |
| Participants without known chronic diseases at baseline, adjusted for smoking status, and excluding the first 3 years of follow-up (primary prespecified analysis) | | | | | | | |
| Participants/deaths | 16701/1440 | 36957/2220 | 114513/6003 | 126914/5878 | 88193/4112 | 39922/1940 | 17563/972 |
| Deaths/PYs^a^ (/1000) | 9.29 | 7.69 | 7.74 | 7.15 | 7.19 | 7.58 | 8.80 |
| HRs (95% CIs) | 1.22 (1.15-1.30) | 1.04 (0.99-1.09) | 1.06 (1.03-1.10) | 1.00 | 1.02 (0.98-1.06) | 1.06 (1.01-1.12) | 1.22 (1.14-1.31) |
| Never-smokers without known chronic diseases at baseline, excluding the first 3 years of follow-up | | | | | | | |
| Participants/deaths | 10354/620 | 21524/920 | 70419/2680 | 81426/2917 | 56428/2212 | 26190/1129 | 12597/651 |
| Deaths/PYs^a^ (/1000) | 6.59 | 5.81 | 5.83 | 5.47 | 5.74 | 6.01 | 7.09 |
| HRs (95% CIs) | 1.15 (1.05-1.25) | 1.03 (0.95-1.11) | 1.05 (1.00-1.11) | 1.00 | 1.05 (0.99-1.11) | 1.09 (1.02-1.17) | 1.28 (1.17-1.39) |

HR = hazard ratio; CI = confidence interval; BMI = body-mass index; PYs = person years.

^a^Adjusted for age, sex, and study area.

Multivariable model was stratified by age, sex, and study area and adjusted for the same set of covariates as in the Table 2.

**Table S5. Adjusted HRs (95% CIs) for association between BMI and sepsis-related mortality with adjustment for WC**

| **BMI (kg/m^2^)** | **<18.5** | **18.5 to <20.0** | **20.0 to <22.5** | **22.5 to <25.0** | **25.0 to <27.5** | **27.5 to <30.0** | **≥30.0** |
| --- | --- | --- | --- | --- | --- | --- | --- |
| Primary analysis^a^ | 2.42 (2.07-2.84) | 1.59 (1.36-1.85) | 1.21 (1.06-1.38) | 1.00 | 0.97 (0.83-1.13) | 0.98 (0.80-1.21) | 1.22 (0.93-1.60) |
| + Further adjusted for WC | 2.99 (2.42-3.69) | 1.85 (1.54-2.23) | 1.32 (1.14-1.52) | 1.00 | 0.90 (0.76-1.06) | 0.85 (0.67-1.06) | 0.96 (0.70-1.32) |

HR = hazard ratio; CI = confidence interval; BMI = body-mass index; WC = waist circumference.

^a^Primary analysis was conducted among participants without known chronic diseases at baseline and excluding the first 3 years of follow-up. Multivariable model was stratified by age, sex, and study area and adjusted for the same set of covariates as in the Table 2.

**Table S6. Adjusted HRs (95% CIs) for association between WC and sepsis-related mortality with adjustment for BMI**

| **WC, quintile** | **Q1** | **Q2** | **Q3** | **Q4** | **Q5** |
| --- | --- | --- | --- | --- | --- |
| Primary analysis^a^ | 1.19 (1.03-1.39) | 0.99 (0.85-1.15) | 1.00 | 0.96 (0.82-1.13) | 1.26 (1.07-1.50) |
| + Further adjusted for BMI | 1.01 (0.86-1.19) | 0.92 (0.78-1.07) | 1.00 | 1.04 (0.88-1.22) | 1.54 (1.28-1.84) |

HR = hazard ratio; CI = confidence interval; BMI = body-mass index; WC = waist circumference.

^a^Primary analysis was conducted among participants without known chronic diseases at baseline and excluding the first 3 years of follow-up. Multivariable model was stratified by age, sex, and study area and adjusted for the same set of covariates as in the Table 2 and hip circumference.


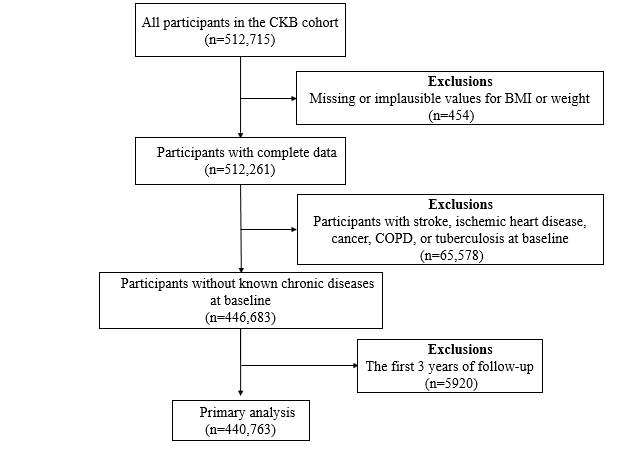


**Figure S1. Flow diagram for study participants in the primary analysis**


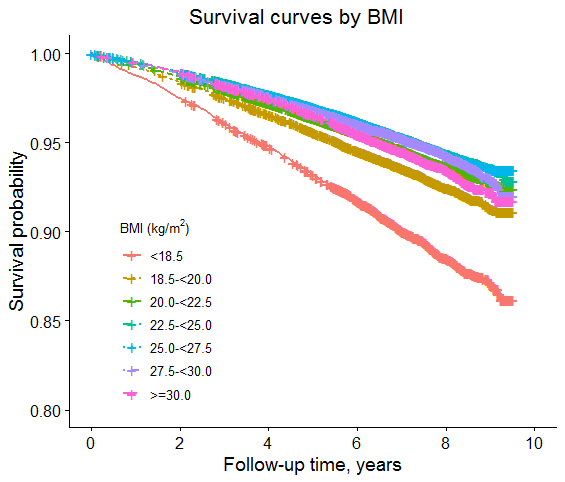


**Figure S2. Kaplan Meier survival probabilities by BMI**

BMI = body-mass index.


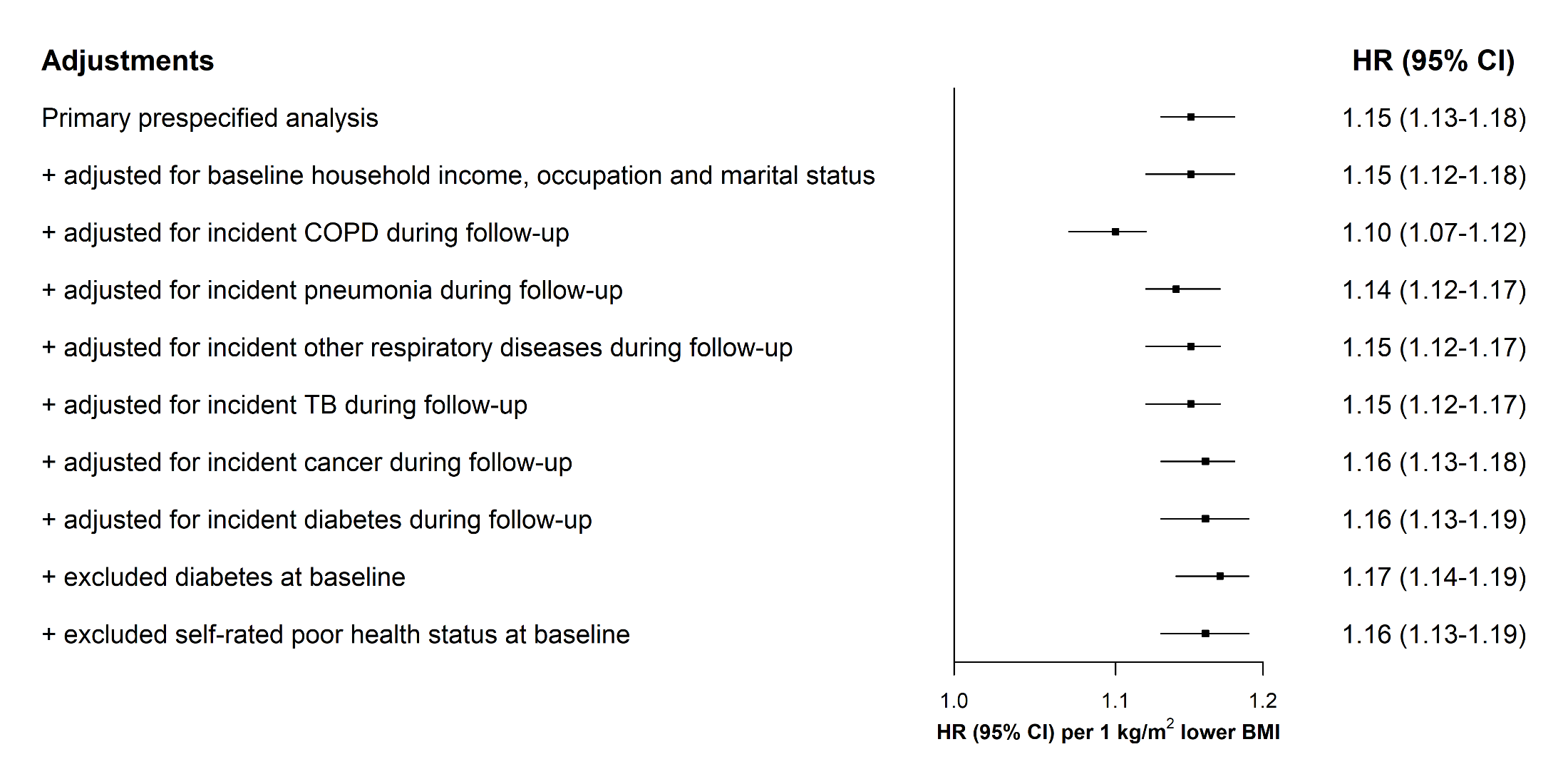
**Figure S3. Adjusted HRs per 1 kg/ m^2^ lower BMI for sepsis-related mortality at BMI <25 kg/m^2^ by applying additional adjustments or exclusions**

HR = hazard ratio; CI = confidence interval; BMI = body-mass index; COPD = chronic obstructive pulmonary disease; TB = tuberculosis.

Primary analysis was conducted among participants without known chronic diseases at baseline and excluded the first 3 years of follow-up. Multivariable model was stratified by age, sex, and study area and adjusted for the same set of covariates as in the Table 2.


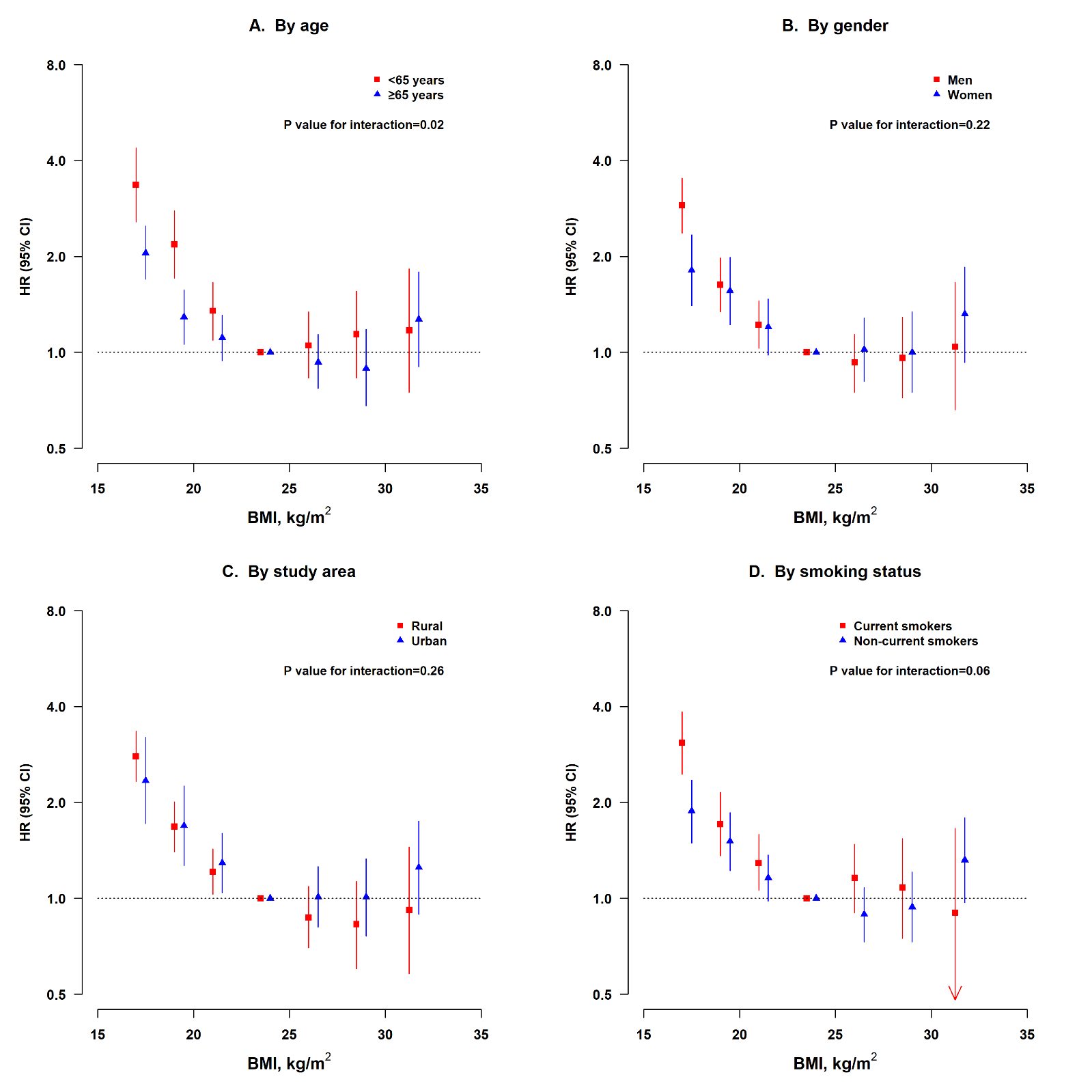
**Figure S4. Association between BMI and sepsis-related mortality by baseline factors**

HR = hazard ratio; CI = confidence interval; BMI = body-mass index.

Subgroup analyses were conducted among participants without known chronic diseases at baseline and excluded the first 3 years of follow-up. Multivariable model was stratified by age, sex, and study area as appropriate and adjusted for the same set of covariates as in the Table 2.
